# Establishment of optimal mandibular incisor inclination: A retrospective comparison between surgery-first and conventional orthodontic approaches

**DOI:** 10.1371/journal.pone.0341798

**Published:** 2026-03-06

**Authors:** Sang-Min Jeon, KyungMin Clara Lee

**Affiliations:** 1 Private Practice, Gwangju, Korea; 2 Department of Orthodontics, Chonnam National University School of Dentistry, Gwangju, Korea; Universidade Federal Fluminense, BRAZIL

## Abstract

**Introduction:**

Surgery-first orthodontic treatment offers early facial profile improvement and shorter treatment duration. This study investigated the incisor relationship in skeletal Class III malocclusion patients treated with conventional orthognathic surgery (COS) and the surgery-first approach (SFA).

**Materials and methods:**

Sixty-eight patients who underwent mandibular setback surgery for skeletal Class III malocclusion were divided into 2 groups: the COS group (n = 34, 19 males and 15 females; mean age, 20.5 ± 2.9 years) treated with conventional three-stage surgical orthodontic treatment and the SFA group (n = 34, 20 males and 14 females; mean age, 21.9 ± 3.5 years) treated without presurgical orthodontics. Lateral cephalograms were analyzed before treatment (T0) and after debonding (T1). The changing pattern of mandibular incisor mandibular plane angle (IMPA) was compared between the groups, and the amount of change was assessed using analysis of covariance (ANCOVA) and mandibular incisor inclinations at each stage were compared to the norm value to calculate the achievement ratio.

**Results:**

At T0, the IMPA was 81.6° in the COS group and 80.7° in the SFA group. At T1, the IMPA was 88.6° in the COS group and 83.3° in the SFA group. Considerable presurgical decompensation occurred in the COS group, while postsurgical decompensation was insufficient in the SFA group. ANCOVA revealed that the COS group exhibited a significantly greater change in IMPA than the SFA group (p = 0.001). At T1, the achievement ratio showed that the COS group (94.4%) was closer to the norm than the SFA group (85.5%) (p = 0.001), indicating that the SFA group showed more compensated mandibular incisors than the COS group.

**Conclusion:**

The COS group demonstrated more effective mandibular incisor decompensation and achieved values closer to the norm than the SFA group. These findings suggest that presurgical orthodontics is essential for achieving optimal incisor inclination, and clinicians should carefully consider the need for sufficient decompensation when planning treatment with the SFA approach.

## Introduction

Skeletal Class III malocclusion, often characterized by mandibular prognathism, commonly presents with labial inclination of the maxillary incisors and lingual inclination of the mandibular incisors due to dental compensation [1]. The primary goal of presurgical orthodontic treatment in such cases is to decompensate the maxillary and mandibular dentition and establish proper arch coordination [2,3]. For instance, when there is significant maxillary incisor proclination relative to the occlusal plane, maxillary premolar extraction may be employed to facilitate effective decompensation. In contrast, mandibular premolar extraction is generally avoided. Additional strategies, such as flattening the curve of Spee, promoting spontaneous alignment, and applying active labial crown torque, are often utilized to achieve proper mandibular incisor decompensation. Successful decompensation is essential for enabling sufficient surgical movement and ensuring optimal postoperative occlusion and stability.

Traditionally, orthognathic treatment follows a three-stage sequence consisting of presurgical orthodontic treatment, surgery, and postsurgical orthodontic treatment. This conventional orthognathic surgery (COS) approach allows clinicians to achieve decompensation before surgery, which facilitates more ideal skeletal correction. In contrast, the surgery-first approach (SFA) omits the presurgical orthodontic phase and proceeds directly to surgery, with orthodontic treatment carried out entirely afterward [4–6]. While SFA offers benefits such as early facial profile improvement and reduced overall treatment time, the absence of a presurgical decompensation phase raises concerns regarding the adequacy of dental compensation, particularly for the mandibular incisors, which may be more difficult to upright after surgery.

Given these differences, it is reasonable to expect that the extent and timing of mandibular incisor decompensation will differ between the two treatment approaches. However, there is a lack of research directly comparing the final outcomes of incisor inclination and decompensation in COS and SFA protocols. In this study, only patients who underwent pure SFA—without any presurgical orthodontic procedures—were included to clearly evaluate the true skeletal and dental effects of the surgery-first concept. A comparable sample of COS patients was also selected to match the skeletal characteristics and treatment goals. To minimize the influence of confounding factors and intergroup variability, analysis of covariance (ANCOVA) was employed, accounting for collinearity among relevant variables. Furthermore, treatment efficacy was assessed by analyzing how mandibular incisor inclination changed during the overall treatment period and by comparing the final inclination outcomes between the two approaches. Therefore, this study aimed to investigate and compare mandibular incisor inclination and the degree of decompensation in skeletal Class III malocclusion patients treated with either COS or the SFA. The null hypothesis was that there would be no significant difference in the final mandibular incisor inclination or in the pattern of inclination change between the two treatment approaches.

## Materials and methods

This retrospective study was approved by the Institutional Review Board (IRB) of the Chonnam National University Dental Hospital, Korea. Medical records of patients diagnosed with skeletal Class III malocclusion and treated with isolated mandibular setback surgery were retrospectively reviewed. As this study involved a retrospective review of anonymized patient data, the requirement for informed consent was waived by the IRB. This retrospective observational study was conducted in accordance with the STROBE (Strengthening the Reporting of Observational Studies in Epidemiology) guidelines to ensure comprehensive and transparent reporting. The data were accessed for research purposes from October 3, 2018 to October 2, 2019. The author did not have access to personally identifiable information during or after data collection. Patients were selected based on the following inclusion criteria: (1) skeletal Class III malocclusion, (2) ANB < 0°, and (3) lateral cephalograms and cone-beam computed tomography images (CBCT) obtained at each treatment stage, before treatment (T0) and after treatment (T1). Exclusion criteria were: presence of cleft lip/palate or other craniofacial syndromes, severe facial asymmetry (≥ 5 mm of chin point deviation from the facial midline), congenitally missing tooth in the anterior region, or tooth anomaly, and previous orthodontic treatment. After applying the inclusion and exclusion criteria, a total of 68 patients with skeletal Class III malocclusion who underwent surgical orthodontic treatment with isolated mandibular setback surgery were finally included and divided into two groups: 1) COS (n = 34) treated with presurgical orthodontic treatment, orthognathic surgery, and postsurgical orthodontic treatment and 2) SFA (n = 34) treated with orthognathic surgery and postsurgical orthodontic treatment. The average treatment period was 27.7 ± 3.8 months in the COS group and 18.7 ± 3.6 months in the SFA group. The demographic data of the two groups are shown in Table 1.

**Table 1:** Demographic data of the patients.

*Variables*	*COS group* *(n = 34)*		*SFA group* *(n = 34)*		*P value*
	*Mean*	*SD*	*Mean*	*SD*	
Age (year)	20.5	2.9	21.9	3.5	0.247
Treatment duration (month)					
Preoperative orthodontic treatment	17.4	2.2	_	_	_
Postoperative orthodontic treatment	10.3	2.7	18.7	3.6	0.005^*^
Total treatment	27.7	3.8	18.7	3.6	0.001^*^
Amount of crowding (mm)					
Maxillary arch	1.9	2.1	1.0	1.8	0.084
Mandibular arch	2.2	1.7	1.8	2.4	0.132

COS group, Conventional orthognathic surgery; SFA group, surgery-first approach. Independent *t-*test was performed to compare the variables between the two groups. ^*^P < 0.01.

**Fig 1:**
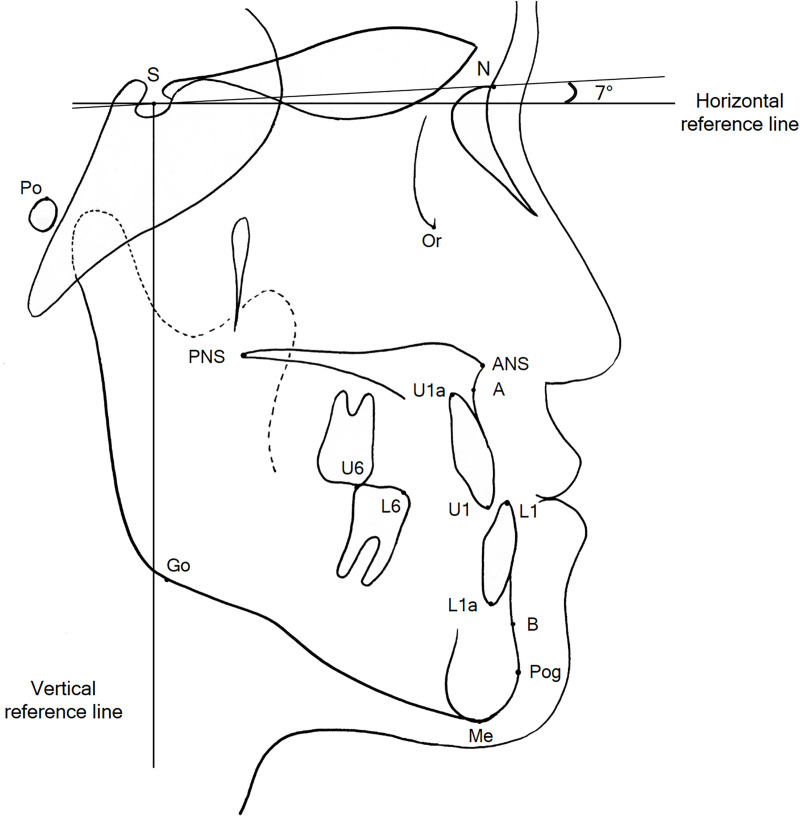
Landmarks and reference planes. S, Sella; N, nasion; Or, orbitale; Po, porion; A, Point A; ANS, anterior nasal spine; PNS, posterior nasal spine; B, Point B; Pog, pogonion; Me, menton; Go, gonion; U1, incisal edge of the upper central incisor; U1a, root apex of U1; L1, incisal edge of the lower central incisor; L1a, root apex of L1; U6, mesiobuccal cusp tip of the upper first molar; L6, MBC of the lower first molar; horizontal reference line, angulated 7° clockwise to the SN line passing through sella; vertical reference line, a perpendicular line to the horizontal reference line passing through sella.

**Fig 2:**
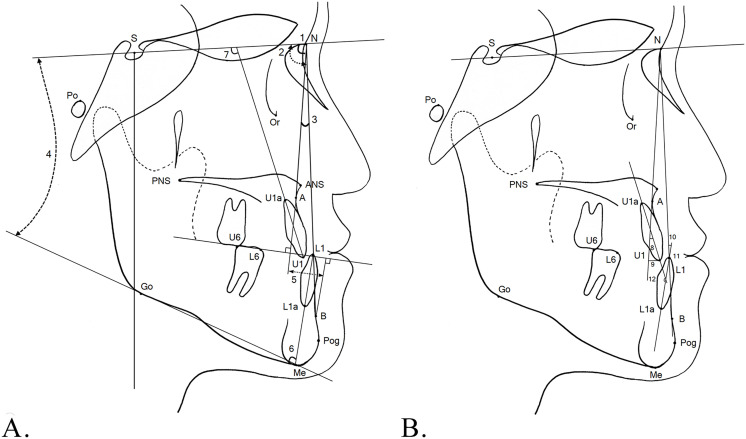
Cephalometric variables: 1, SNA (°); 2, SNB (°); 3, ANB (°); 4, SN/GoGn (°); 5, Wits appraisal (mm); 6, IMPA (°); 7, U1 to SN (°); 8, U1 to NA (°); 9, U1 to NA (mm); 10, L1 to NB (°); 11, L1 to NB (mm); 12, Interincisal angle (°).

**Fig 3:**
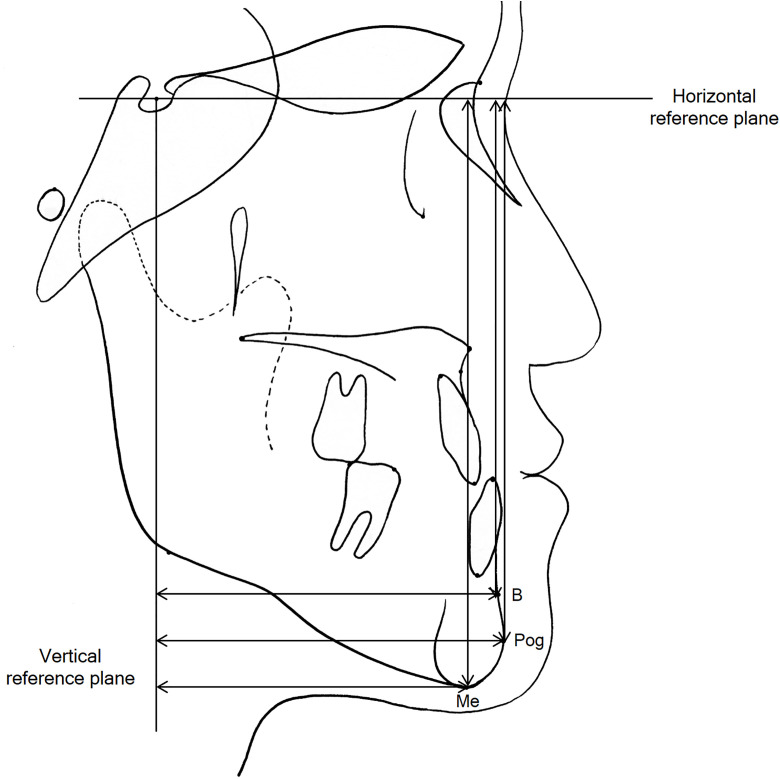
Measurements of the amount of surgical movement of the mandible.

All patients were treated with a 0.018-inch straight wire appliance with the Roth prescription and sliding mechanics. The study used 0.016 × 0.022-in stainless steel wires as surgical and final archwires. Class II elastics for decompensation or anchorage reinforcement, such as transpalatal arch and mini-implants, were not used during presurgical orthodontic treatment. Postsurgical orthodontic treatment started after 3 weeks of surgical wafer wear. The mechanics of postsurgical orthodontic treatment did not differ between the two groups. Lateral cephalograms were taken before treatment (T0) and after treatment at debonding (T1). Definitions of the landmarks and reference planes are shown in Fig 1. The cephalograms were digitized by a single examiner using the V-Ceph program (version 8.0, CyberMed, Seoul, Korea). Cephalometric skeletodental variables are shown in Fig 2. To assess the amounts of surgical movement of the mandible, the horizontal and vertical distances from Point B, pogonion, and menton to the vertical and horizontal reference lines were obtained from the lateral cephalograms taken at T0 and T1 (Fig 3). The horizontal reference line was set to be angulated 7° clockwise to the Sella-Nasion line passing through the sella. The vertical reference plane was perpendicular to the horizontal reference line passing through the sella.

The amount and pattern of change in the incisor mandibular plane angle (IMPA) were calculated to assess treatment efficacy. Treatment efficacy was defined as the extent to which the mandibular incisor inclination was corrected toward the normative angulation. To evaluate this, the achievement ratio was determined by dividing the actual change in IMPA achieved during treatment by the expected change in IMPA required to reach the ideal surgical objective. In addition, differences in the pattern of IMPA change between the two groups were analyzed.

### Statistical analysis

Statistical evaluations were performed at a 5% significance level using SPSS software (version 29.0, IBM, Armonk, NY). Sample size calculation for ANCOVA was based on the result of a previous study by Kee et al. [7]. This study used secondary data obtained from the previous research [7]. The earlier study investigated different outcome variables, whereas the present analysis focused specifically on mandibular incisor inclination and decompensation patterns between the COS and SFA groups. To evaluate the effect of an intervention (COS vs. SFA) on IMPA before and after treatment (2 covariates), an expected effect of medium size 0.5, a statistical power of 80%, a type I error of 5%, numerator degrees of freedom = 1, number of groups = 4, and number of covariates = 2 were assumed by the G*power program (version 3.1.9.2, Heinrich-Heine-University, Dusseldorf, Germany). The calculation indicated 34 patients to be included in each group.

The values of cephalometric variables were obtained at T0 and T1. The measurements were initially tested for normal distribution and were subjected to paired *t-*tests in the COS and SFA groups to determine potential statistically significant differences. ANCOVA was performed to analyze the differences in the pattern of change in IMPA between the two groups, and a paired *t*-test was used within each group according to the treatment.

All measurements were obtained by a single examiner who repeated the measurements from 20 randomly selected patients for intrarater reliability after 2 weeks. Differences calculated with Dahlberg’s formula [8] ranged from 0.15 to 0.20 mm for linear measurements and 0.25° to 0.30° for angular measurements. The intracorrelation coefficient (ICC) values ranged from 0.77 to 0.90, with a mean of 0.83, and indicated excellent reliability.

## Results

There were no statistically significant differences in the amount of surgical movement of the mandible between COS and SFA groups (Table 2). As for the cephalometric variables pre- and post-treatment in each COS and SFA group, IMPA, U1 to SN, U1 to NA (°), L1 to NB (°), L1 to NB (mm), and interincisal angles at post-treatment showed statistically significant differences between COS and SFA groups (Table 3). The IMPA, U1 to SN, L1 to NB (°), and L1 to NB (mm)showed no statistically significant differences between the two groups before treatment but exhibited statistically significant differences between the two groups after treatment (Table 3). At T0, IMPA was 81.6° in the COS group and 80.7° in the SFA group. At T1, IMPA was 88.6° in the COS group and 83.3° in the SFA group. After treatment, the U1 to SN increased by an average of 1.9° in the COS group and 1.1° in the SFA group, showing a difference between the groups. The U1 to NA (°) increased by an average of 0.9° in the COS group and 1.2° in the SFA group, also indicating a difference between the groups. For L1 to NB (°), the COS group showed an average increase of 3.8°, whereas the SFA group showed an increase of 2.1°, again demonstrating a difference between the groups. The L1 to NB (mm) increased by an average of 1.2 mm in the COS group and 0.6 mm in the SFA group. The interincisal angle decreased by an average of 8.6° in the COS group and 7.2° in the SFA group. Considerable postsurgical decompensation occurred in the COS group, while insufficient postsurgical decompensation occurred in the SFA group. The SFA group exhibited more compensated mandibular incisors than the COS group (Table 3).

**Table 2:** Comparison of the amount of surgical movement of the mandible.

	*COS group* *(n = 34)*		*SFA group* *(n = 34)*		*P value*
	*Mean*	*SD*	*Mean*	*SD*	
Horizontal movement (mm)					
∆ B (x)	−6.9	3.4	−7.4	3.2	0.982
∆ Pog (x)	−6.8	3.5	−8.1	3.8	0.591
∆ Me (x)	−6.1	4.0	−7.2	3.3	0.408
Vertical movement (mm)					
∆ B (y)	0.6	2.5	0.83	3.3	0.904
∆ Pog (y)	0.3	1.8	−0.19	1.9	0.177
∆ Me (y)	0.4	1.5	0.83	1.6	0.314

COS group, Conventional orthognathic surgery; SFA group, surgery-first approach; SD, standard deviation. Independent *t-*test was performed to compare the variables between the two groups. For the horizontal movement, (-) indicates setback. For the vertical movement, (-) indicates superior impaction; (+) indicates inferior elongation.

**Table 3:** Comparison of the cephalometric variables pre- and post-treatment in each group.

*Variables*	*Norm* ^¶^	*Pretreatment*					*Posttreatment*				
		*COS group* *(n = 34)*		*SFA group* *(n = 34)*		*P value*	*COS group* *(n = 34)*		*SFA group* *(n = 34)*		*P value*
		*Mean*	*SD*	*Mean*	*SD*		*Mean*	*SD*	*Mean*	*SD*	
SNA (°)	81.3	81.5	2.7	81.7	4.1	0.788	81.4	2.7	81.6	4.3	0.902
SNB (°)	78.9	84.4	3.7	84.8	4.6	0.715	80.5	2.6	80.9	4.3	0.690
ANB (°)	2.6	−2.9	2.1	−3.1	2.5	0.802	0.9	1.2	0.8	1.6	0.817
SN/GoGn (°)	33.8	34.2	5.3	33.2	6.4	0.568	36.9	5.5	36.5	6.0	0.817
Wits appraisal (mm)	−1.7	−11.3	3.6	−12.0	4.8	0.583	−2.9	2.1	−3.2	2.7	0.707
IMPA (°)	95.4	81.6	6.8	80.7	7.3	0.562	88.6	4.2	83.3	5.7	0.036^*^
U1 to SN (°)	106.6	111.7	5.6	108.3	6.6	0.052	113.6	5.8	109.4	6.8	0.026^*^
U1 to NA (°)	29.1	31.1	5.5	26.6	5.9	0.007^*^	32.0	5.4	27.8	5.9	0.012^*^
U1 to NA (mm)	6.3	9.3	2.2	7.7	2.8	0.034^*^	8.7	1.8	8.1	2.4	0.282
L1 to NB (°)	25.3	20.4	5.7	18.8	7.1	0.383	24.2	5.0	20.9	5.5	0.030^*^
L1 to NB (mm)	6.0	6.2	2.7	5.5	2.3	0.337	7.4	1.9	6.1	2.4	0.044^*^
Interincisal angle (°)	127.1	131.5	9.7	137.7	10.2	0.030^*^	122.9	8.4	130.5	6.6	0.011^*^
Overjet (mm)	3.6	−1.9	2.2	−2.8	2.3	0.192	2.2	1.1	2.2	0.7	0.688
Overbite (mm)	1.5	1.0	2.0	1.4	2.4	0.499	1.7	0.9	1.9	0.7	0.420
Curve of Spee (mm)	1.5	2.3	0.6	2.1	0.4	0.165	1.6	0.5	2.0	0.4	0.083

*COS*, conventional orthognathic surgery; and *SFA*, surgery-first approach. ^¶^ The ethnic norms [9,10] *SD,* standard deviation. Independent *t*-test was performed to compare the variables between the two groups at each stage. ^*^P < 0.05.

**Fig 4:**
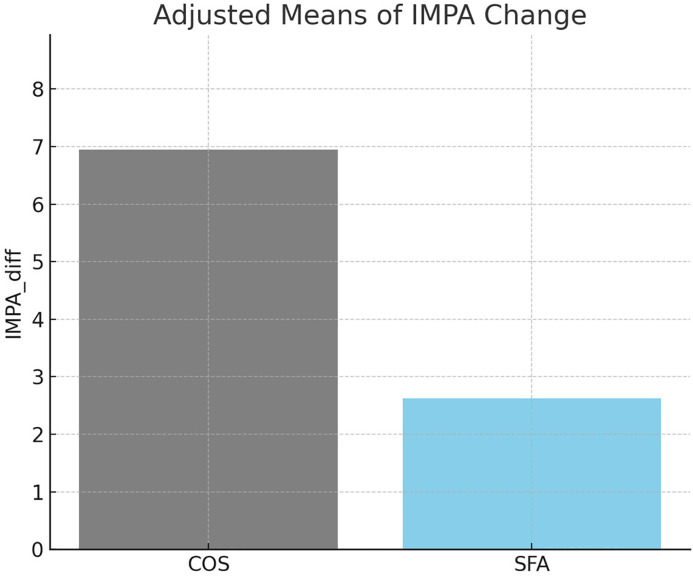
Comparison of the changing pattern of the IMPA according to the treatment in each group. ANCOVA was used to analyze the differences in the changing pattern of the IMPA between the two groups.

ANCOVA was conducted to compare the amount of change in IMPA between the COS and SFA groups, controlling for their respective baseline values (Table 4). ANCOVA revealed a statistically significant difference between the two groups after adjusting for baseline IMPA values (F = 16.54, p < 0.001). The COS group demonstrated a significantly greater improvement in mandibular incisor inclination compared to the SFA group. The adjusted mean change in IMPA was higher in the COS group (6.9°) than in the SFA group (2.6°) (Fig 4).

**Table 4:** Comparison of changing patterns according to treatment in IMPA between COS and SFA groups.

*Variable*	*COS group* *(n = 34)*		*SFA group* *(n = 34)*		*P value*
	*Mean*	*SD*	*Mean*	*SD*	
IMPA	6.9	4.7	2.6	5.8	0.001^*^

COS, Conventional orthognathic surgery; SFA, surgery-first approach; SD, standard deviation. *p *<* 0.01 by ANCOVA.

Table 5 shows the pre- and post-treatment changes in the relative and achievement ratios of IMPA between the COS and SFA groups. The relative ratio to the norm [9,10] of IMPA at T0 was 85.6% in the COS group and 84.5% in the SFA group. There was no statistically significant difference in the relative ratio to the norm of IMPA between the COS and SFA groups at T0. However, after treatment, there were statistically significant differences in the relative ratio to the norm and the achievement ratio of IMPA between the two groups. The relative ratio to the norm of IMPA at T1 was 92.9% in the COS group and 87.3% in the SFA group. A comparison of the achievement ratio showed that the IMPA value in the COS group (94.4%) was statistically closer to the norm than that in the SFA group (85.5%). Regarding treatment efficacy, the COS group showed a closer to the norm and a higher achievement ratio than the SFA group after treatment.

**Table 5:** Efficacy in terms of relative ratio to Norm and achievement ratio of mandibular incisor inclination (IMPA).

	*COS group* *(n = 34)*		*SFA group* *(n = 34)*		*P value*
	*Mean*	*SD*	*Mean*	*SD*	
Relative ratio to Norm (%)					
Before treatment (T0)	85.6	7.1	84.5	7.7	0.620
After treatment (T1)	92.9	4.4	87.3	6.0	0.001^*^
Achievement ratio (%)	94.4	3.3	85.5	6.6	0.001^*^

COS, Conventional orthognathic surgery; SFA, surgery-first approach. The relative ratio to the ethnic norm of IMPA (95.4°) [9,10] is the (actual value of IMPA/95.4°). The achievement ratio is (the actual amount of changes in IMPA with treatment/expected amount of changes in IMPA for the surgical treatment objective). ^*^P < 0.01 by paired *t*-*t*est.

## Discussion

Presurgical orthodontic decompensation ideally requires coordinated correction in the sagittal, transverse, and vertical dimensions [11–13]. However, evaluating transverse or vertical changes is often confounded by treatment decisions such as maxillary premolar extraction, arch form modification, and occlusal plane alteration. To minimize these confounding factors, the present study focused specifically on the sagittal decompensation of the mandibular incisors. This approach ensured a more controlled interpretation of the primary variable (IMPA) while other planes of decompensation are being addressed in ongoing companion studies. Previous reports have emphasized that sagittal decompensation of the mandibular incisors is a key determinant of achieving stable anteroposterior jaw correction in Class III patients [14–16].

The primary objectives of presurgical orthodontics in skeletal Class III malocclusion are to decompensate the dentition, coordinate the arches, and prepare an optimal occlusal environment for orthognathic surgery [11–14]. Skeletal discrepancies elicit dentoalveolar compensations, and the magnitude of these compensations increases with the severity of the skeletal disharmony [11–14]. Among them, the inclination of the mandibular incisors is strongly influenced by the available labial and lingual alveolar housing [17,18]. Several CBCT-based studies have demonstrated that limitations in alveolar bone thickness restrict the safe extent of retroclination or proclination, thereby influencing both presurgical objectives and post-treatment stability [7,19–21]. These anatomical constraints must be acknowledged when interpreting IMPA changes, as they determine the biologically feasible range of decompensation.

In addition to alveolar bone morphology, the inclination of the occlusal plane significantly affects the interpretation of mandibular incisor angulation. A steepening occlusal plane can create the appearance of increased incisor proclination, whereas a flattening plane may mask true angular changes [22,23]. Because IMPA is measured relative to the mandibular plane, its value is influenced not only by tooth inclination but also by skeletal rotational patterns. In the present study, both groups exhibited an increase in the SN–GoGn angle after surgery. This suggests that vertical mandibular rotation—likely secondary to surgical autorotation, condylar seating changes, or neuromuscular adaptation—may have contributed to the measured differences in IMPA. These vertical changes highlight the limitation of interpreting IMPA as a stand-alone indicator and reinforce the need to consider occlusal and skeletal factors simultaneously.

The results demonstrated that the COS group achieved a closer approximation to the normative IMPA and exhibited a higher achievement ratio compared with the SFA group. These findings reflect the inherent differences between conventional and surgery-first protocols. Because SFA bypasses presurgical decompensation, the opportunity for mandibular incisor correction is deferred to the postsurgical phase. However, once the surgical correction establishes the overjet, the available space for dental decompensation becomes limited and may not meet the requirements for ideal incisor inclination correction [24,25]. Furthermore, the postsurgical period presents additional biomechanical challenges, including muscular adaptation and potential mandibular relapse, particularly in the horizontal dimension [26,27]. These factors may collectively contribute to the reduced treatment efficacy observed in the SFA group. The incorporation of temporary anchorage devices (TAD)s may enhance postsurgical decompensation, but the predictability and extent of achievable correction remain inherently more limited than in presurgical orthodontic protocols [24,25].

The COS and SFA groups showed statistically significant differences in the relative ratio to the norm and the achievement ratio of IMPA after treatment. Regarding treatment efficacy, the COS group showed a closer to the norm and a higher achievement ratio than the SFA group after treatment, indicating that the COS group achieved better outcomes for IMPA than the SFA group. SFA treatment does not involve presurgical orthodontic treatment with decompensation, which may result in reduced efficacy of mandibular incisor decompensation. Since the SN/Go-Gn angle increased in both groups after surgery, changes in mandibular plane inclination may have partially influenced the observed differences in IMPA. Therefore, the evaluation of mandibular incisor inclination should be interpreted in relation to the overall occlusal plane and mandibular plane changes rather than as an isolated value. In this study, treatment efficacy was defined as the extent to which the mandibular incisor inclination approached the normative angulation. However, changes in the occlusal plane and mandibular plane were also taken into account when interpreting these outcomes. Further studies with a more comprehensive three-dimensional assessment are needed to clarify these relationships.

In the SFA approach, decompensation must be completed after surgery; however, in clinical situations, it can be challenging to achieve sufficient decompensation because the overjet has already been determined by the surgical correction. Therefore, the surgical plan should carefully calculate and incorporate the necessary skeletal correction to secure adequate overjet immediately after surgery, facilitating effective postsurgical decompensation. In such cases, the use of TADs should also be considered to support the required tooth movement during the postsurgical phase [24,25]. Despite these strategies, it remains difficult to precisely predict and manage potential postsurgical mandibular relapse, especially in the horizontal dimension [26–30]. Consequently, insufficient overjet is available for dental decompensation during postsurgical orthodontic treatment. Therefore, the mechanism for decompensatory tooth movement is more complex in postsurgical orthodontic treatment than in presurgical orthodontic treatment.

### Limitation

This study has limitations inherent to its retrospective design, including potential bias in sample selection and constraints in determining the optimal sample size. Although strict inclusion and exclusion criteria were applied to both the COS and SFA groups to minimize such bias, the possibility of unrecognized confounding factors cannot be fully eliminated. Future prospective studies with larger and more diverse samples are therefore needed to validate and expand upon the present findings. In addition, while IMPA served as the primary indicator of decompensation in this study, its interpretation should be considered within the broader anatomical and biomechanical context, including alveolar bone limitations, occlusal plane inclination, and vertical skeletal rotation. Future research incorporating three-dimensional analyses—such as CBCT-based alveolar bone mapping, occlusal plane evaluation, and detailed assessment of mandibular rotation—will provide a more comprehensive understanding of how these interacting factors influence decompensation outcomes and long-term stability.

## Conclusion

Patients treated with SFA exhibited more compensated mandibular incisors than those in the COS group after treatment, indicating that pre-surgical orthodontics is more effective in achieving proper decompensation of the mandibular incisors. Therefore, clinicians should carefully consider the need for sufficient mandibular incisor decompensation during the postsurgical phase when using the SFA approach.
